# PaPI: pseudo amino acid composition to score human protein-coding variants

**DOI:** 10.1186/s12859-015-0554-8

**Published:** 2015-04-19

**Authors:** Ivan Limongelli, Simone Marini, Riccardo Bellazzi

**Affiliations:** 1 IRCCS Policlinico S. Matteo, Pzz.le Volontari del Sangue 2, 27100 Pavia, Italy; 2 Department of Electrical, Computer and Biomedical Engineering, University of Pavia, Via Ferrata 1, 27100 Pavia, Italy

**Keywords:** Sequencing, Variant, Prediction, Exome, Pseudo amino acid composition

## Abstract

**Background:**

High throughput sequencing technologies are able to identify the whole genomic variation of an individual. Gene-targeted and whole-exome experiments are mainly focused on coding sequence variants related to a single or multiple nucleotides. The analysis of the biological significance of this multitude of genomic variant is challenging and computational demanding.

**Results:**

We present PaPI, a new machine-learning approach to classify and score human coding variants by estimating the probability to damage their protein-related function. The novelty of this approach consists in using pseudo amino acid composition through which wild and mutated protein sequences are represented in a discrete model. A machine learning classifier has been trained on a set of known deleterious and benign coding variants with the aim to score unobserved variants by taking into account hidden sequence patterns in human genome potentially leading to diseases. We show how the combination of amphiphilic pseudo amino acid composition, evolutionary conservation and homologous proteins based methods outperforms several prediction algorithms and it is also able to score complex variants such as deletions, insertions and indels.

**Conclusions:**

This paper describes a machine-learning approach to predict the deleteriousness of human coding variants. A freely available web application (http://papi.unipv.it) has been developed with the presented method, able to score up to thousands variants in a single run.

**Electronic supplementary material:**

The online version of this article (doi:10.1186/s12859-015-0554-8) contains supplementary material, which is available to authorized users.

## Background

New sequencing technologies are becoming increasingly cost effective [1-3]. At the same time, human genomic data availability is expected to increase due to the growing number of human genomes being sequenced. As a matter of fact, targeted sequencing and whole-exome sequencing have become nowadays well-established strategies to identify genomic variants related to diseases and drastically reduce sequencing costs [4-8].

One of the major challenges for these kinds of studies consists to point out, among the multitude of the identified variants, those that are related to a target phenotype or are potentially harmful for the individual health.

A basic approach consists of checking whether a variant is reported and described in one of the publicly available resources, e.g. the 1000 Genomes Project database (1TGP) [9] with 1092 individual human genome sequences along with their coding and noncoding variants, and the NHLBI GO Exome Sequencing Project (ESP) [10], reporting only exome variants from 6503 human samples across diverse populations. Phenotype-related genomic variants resources are also available, such as ClinVar [11] and Human Gene Mutation Database (HGMD) [12], the latter made of coding variants in the 75% of cases.

Unfortunately, only a small number of variants of the human genome have been identified as pathogenic by family or cohort studies and clinically validated by experimental evidence [13].

For this reason, several algorithms have been developed in order to score variants according to the inferred deleteriousness for their encoded protein. They are generally based on four different approaches: multiple sequence alignment (MSA) methods of homologous proteins [14,15] such as SIFT [16], protein structure information such as PolyPhen2 [17], comparative evolutionary data [18-21] and structural or sequence pattern encoding [22]. Since each algorithm has some limitations, one approach to detect a deleterious variant consists of testing several independent methods and checking if at least one assesses its pathogenicity [23,24]. This strategy has high sensitivity, but poor specificity, thus leading to low accuracy. Therefore, a number of algorithms that combine the outputs of several predictors and optimize accuracy on known variant sets have been developed [25-27]. Moreover, methods that use prior knowledge (e.g. Human Phenotype Ontology, Gene Ontology) in combination with functional predictions in order to rank variants on the basis of a given phenotype [28,29] have been successfully implemented, as well.

In this scenario, due to the importance of having more accurate and exhaustive variant functional predictors, we here propose a new phenotype-free method based on pseudo amino acid composition (PseAAC) [30] and evolutionary conservation in combination with other two well-established and commonly-used approaches (PolyPhen2 and SIFT). We believe that our approach may provide a valuable addition to the worldwide research efforts devoted to predicting the role of uncharacterized variants.

PseAAC is a feature encoding method allowing both compositional and positional amino acid pattern representation of peptide primary sequence in a discrete model. Given a peptide sequence, PseAAC is computed by modeling pairwise relationships between amino acids using residues chemical properties (see *Methods*). In particular, we used amphiphilic PseAAC, based on normalized hydrophobicity and hydrophilicity: the arrangement of these two indices along a protein chain play an important role in protein folding, catalytic mechanism and protein interaction with other molecules and environment [31]. For example, hydrophobicity is often a major contributor of binding affinity between a protein and its ligand [32], hydrophilic residues such as Arg, Asp, Lys, and Glu have the highest protein-surface frequencies [33], and intrinsically disordered regions (IDRs) usually have few large hydrophobic residues but favour polar and charged amino acids [34].

Previous studies [35,36] analysed human coding variants in terms of amino acid substitution both in disease and natural background variant datasets, such as HGMD and 1TGP. Such studies showed that disease-associated variant distributions are radically different from neutral amino acid ones and that disease-associated variants exhibit more extreme differences in terms of physicochemical properties such as amino acid volume, charge and hydrophobicity.

We therefore coupled hydrophobicity and hydrophilicity PseAAC feature encoding with machine learning to develop a model able to learn pseudo amino acid composition substitution patterns following coding variants that can alter protein function and/or structure, leading to disease.

The difference in terms of PseAAC between wild and mutated protein sequences together with evolutionary conservation scores of the altered bases have been used as features to train a Random Forest (RF) [37] with the aim to score coding variants into protein-damaging or tolerated class. Since PseAACs model amino acid relationships in terms of hydrophobicity and hydrophilicity arrangements within the wild and mutated sequences, RF is supposed to learn from substitution patterns occurring at amino acid composition level in terms of frequency and order. A variant is therefore implicitly evaluated within its sequence context.

We finally combined the RF output with PolyPhen2 and SIFT by a voting strategy. Despite the advantages of combining PolyPhen2 and SIFT have been previously reported [27], we show the RF inclusion is able to further increase prediction performances.

The overall algorithm, called PaPI, provides predictions even for those variants that the other tools cannot process (e.g. because of lack of data) and it is able to deal with any variant type, including single nucleotide variants and insertion or deletion of several nucleotides.

While RF classifiers have been already used in Genomics, from GWAS to RNA-protein binding prediction [38], to our knowledge, this is the first time that PseAAC is applied to protein variant prediction.

## Results and discussion

Hereby we denote with the term *indel* the following variants: insertions, deletions, insertions followed by deletions (or vice-versa) and multi-nucleotide variants. We refer to single nucleotide variants (SNVs) in case of non-synonymous single nucleotide variants that lead to a single amino acid change. Finally, we denote as *in-frame* and *frameshift* indels those variants causing the insertion/deletion of one or more amino acids and those altering the open reading frame of the coding sequence, respectively.

Known coding disease-related variants (damaging) were retrieved from HGMD, including SNVs and indels. We assumed that frequent genomic variants are less suitable of being deleterious, therefore, tolerated variants were retrieved by combining 1TGP and ESP selecting only polymorphic (frequency higher than 0.05) and unique variants. Due to the unbalancing between damaging versus tolerated variants of the resulting dataset (Table 1), we randomly split it into three quasi-balanced sets. We further split each into a training (70%) and test (30%) set (Table 2). The whole process is explained in the *Methods* section.

**Table 1:** **Damaging and tolerated variant sets**

	**Damaging (HGMD)**	**Tolerated (1TGP + ESP)**
# initial variants	176523	65377
- # SYN	1333	36546
- # FR/SC/SD	77627	929
# final variants	97653	27902

Damaging and tolerated sets after synonymous-SNVs and frameshift, stop-causing and stop-disrupting variants removal. An instance of the data set is the coding variant relative to the transcript to which overlaps. SYN = synonymous, FR = frameshift, SC = stop-causing, SD = stop-disrupting.

**Table 2:** **Three random variant sets**

**Set**	**Training**		**Test**	
	**Damaging**	**Tolerated**	**Damaging**	**Tolerated**
**# 1**	25291	19570	10729	8332
**# 2**	25318	19570	10861	8332
**# 3**	17838	13763	7616	5879

Three quasi-balanced variant sets were generated randomly and divided by training (70%) and test (30%) sets.

For each variant, the difference in PseAAC between wild (reference genome) and mutated amino acid sequence was computed, resulting in a set of quantitative features, used to train and test a machine learning classifier. Three evolutionary conservation scores and three full length protein attributes were included in the feature set as well (see *Methods*).

An RF and a Logistic Regression (LR) models were built upon the resulting training sets, while performances were measured on each relative test set. The RF achieved an average area under the curve (AUC) of 0.897 and an average accuracy of 0.832 on the three sets, resulting in performances higher than the LR ones (AUC = 0.878, accuracy = 0.813, see Table 3 and see Additional file 1: Figure S1). The gap between the two classifiers can be explained by the complexity of the feature set: given its non-linear nature, RF is more suitable to detect hidden structures in data with respect to the LR.

**Table 3:** **Performances of RF and LR on the three test sets**

**Test Set**	**Tool**	**AUC**	**Accuracy [IC95%]**	**Sens**	**Spec**	**PPV**	**NPV**	**F-m**	**MCC**
# 1	RF	.8988	.8314 [.8381-.8246]	.8354	.8274	.8298	.8331	.8326	.6629
	LR	.8770	.8118 [.8188-.8047]	.8410	.7825	.7957	.8301	.8177	.6246
# 2	RF	.90	.8310 [.8377-.8242]	.8370	.8250	.8282	.8340	.8325	.6621
	LR	.8752	.8121 [.8190-8049]	.8464	.7775	.7931	.8340	.8189	.6255
# 3	RF	.9035	.8344 [.8422-8262]	.8406	.8280	.8311	.8377	.8358	.6687
	LR	.8833	.8168 [.8250-.8083]	.8459	.7875	.8003	.8355	.8225	.6346

Performances of the Random Forest (RF) and Logistic Regression (LR) on the three test sets. Area under the curve (AUC), accuracy with 95% confidence interval, sensitivity (Sens), specificity (Spec), Positive Predictive Value (PPV), Negative Predictive Value (NPV), F-measure (F-m) and Matthews correlation coefficient (MCC) are reported for each method.

In order to quantify the contribution of PseAAC features in classification we measured the performance of the RF trained on the aforementioned training sets without evolutionary conservation scores and full length protein features (see Additional file 1: Table S1). In other words, we assessed a RF model based on PseAAC only. Notably, the RF trained solely on PseAAC reached, on average, an AUC and accuracy of 0.88 and 0.82 respectively. This is only about one percent less than the RF holding the complete feature set.

**Figure 1:**
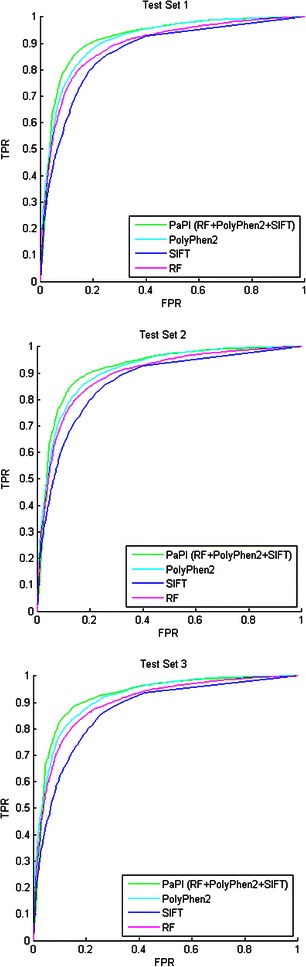
ROC curves of the RF, PolyPhen2, SIFT and PaPI (RF + PolyPhen2 + SIFT) ROC curves of Random Forest (RF), PolyPhen2, SIFT and their ensemble (PaPI) on the three test sets. Variants that PolyPhen2 and/or SIFT were not able to predict were filtered out.

We finally combined the RF model with PolyPhen2 and SIFT scores through the implemented voting scheme. In order to independently measure performances of the three algorithms on the same data, test sets were filtered out for variants that PolyPhen2 and/or SIFT were not able to predict. The combined approach, which we called PaPI, increased the overall performances: AUC, accuracy and Matthews correlation coefficient (MCC) are increased in average by 2, 3 and 7 percentage points respectively when compared to the RF model alone. Sensitivity, specificity and other performance metrics of the RF, PolyPhen2, SIFT and PaPI on the three test set are reported in Table 4 while receiver operating characteristic (ROC) curves are reported in Figure 1. Being PaPI an ensemble method based on three classifiers, we also analysed the prediction consistency among the three tools. The great majority of the correct predictions (over 75%) finds RF, PolyPhen2 and SIFT in agreement. More details are summarized by the Venn diagrams reported see Additional file 1: Figure S2.

**Table 4:** **Performances of RF, PolyPhen2, SIFT and PaPI on the three test sets**

**Test Set**	**Tool**	**AUC**	**Accuracy [IC95%]**	**Sens**	**Spec**	**PPV**	**NPV**	**F-m**	**MCC**
# 1	PaPI	.9207	.8621 [.8553-.8685]	.8580	.8663	.8688	.8553	.8633	.7242
	RF	.8941	.8262 [.8189-.8334]	.8286	.8238	.8291	.8233	.8289	.6524
	PolyPhen2	.9137	.8425 [.8354-.8493]	.8533	.8314	.8392	.846	.8462	.6849
	SIFT	.8682	.8045 [.7968-.812]	.7724	.8376	.8307	.781	.8005	.6108
# 2	PaPI	.9196	.8618 [.8550-.8683]	.8572	.8665	.869	.8545	.8631	.7236
	RF	.8960	.8275 [.8202-.8346]	.8319	.823	.8292	.8257	.8305	.6549
	PolyPhen2	.9121	.8401 [.8330-.847]	.8486	.8314	.8387	.8417	.8436	.6801
	SIFT	.8677	.7994 [.7917-.807]	.7625	.8376	.829	.7735	.7944	.6013
# 3	PaPI	.9239	.8648 [.8568-.8724]	.8570	.8729	.8745	.8553	.8657	.7298
	RF	.8999	.8289 [.8202-.8373]	.8358	.8218	.8289	.8289	.8323	.6577
	PolyPhen2	.9185	.8416 [.8331-.8497]	.8501	.8328	.8401	.8432	.8451	.6831
	SIFT	.8688	.7999 [.7906-.8088]	.7558	.8454	.8348	.7701	.7933	.603

Performances of the Random Forest (RF), PolyPhen2, SIFT and PaPI (RF + PolyPhen2 + SIFT) on the three test. Area under the curve (AUC), accuracy with 95% confidence interval, sensitivity (Sens), specificity (Spec), Positive Predictive Value (PPV), Negative Predictive Value (NPV), F-measure (F-m) and Matthews correlation coefficient (MCC) are reported for each method. Test sets were filtered in order to retain only those variants that both PolyPhen2 and SIFT were able to predict.

### Performances on unpredictable variants for PolyPhen2 and SIFT

We further proceeded to evaluate PaPI performances on those variants of the test sets for which both PolyPhen2 and SIFT were unable to give a prediction, resulting in a total of 416 tolerated and 974 damaging missed variants. In these cases, PaPI classes and scores coincide with the RF predictor ones. The average area under the curve (AUC) of the RF was equal to 0.94 while the average accuracy on the three variant sets was equal to 0.87 (see Table 5 for the performance metrics and Additional file 1: Figure S3 for ROC curves).

**Table 5:** **PaPI performances on the unpredictable variants by PolyPhen2 and SIFT**

**Test Set**	**Tool**	**AUC**	**Accuracy [IC95%]**	**Sens**	**Spec**	**PPV**	**NPV**	**F-m**	**MCC**
# 1	PaPI (RF)	.9368	.8676 [.8420-.8896]	.9171	.8245	.8198	.9196	.8657	.7405
# 2	PaPI (RF)	.9418	.8611 [.8352-8836]	.9214	.8077	.8095	.9205	.8619	.7296
# 3	PaPI (RF)	.942	.8830 [.8523-9080]	.9256	.845	.8421	.9271	.8819	.7699

PaPI performances on the three test retaining only those variants unpredictable both for PolyPhen2 and SIFT. In this case, PaPI coincides with RF. Area under the curve (AUC), accuracy with 95% confidence intervals, sensitivity (Sens), specificity (Spec), Positive Predictive Value (PPV), Negative Predictive Value (NPV), F-measure (F-m) and Matthews correlation coefficient (MCC) are reported for each method.

### Comparison with other variant prediction tools

PaPI’s performances were compared to the following variant predictors: Carol [27], PROVEAN [39], FATHMM [15], MutationAssessor [14], LRT [21], PolyPhen2 and SIFT. A brief description of each algorithm is reported in Additional file 2.

Thanks to the RF model, PaPI is capable to score variants of any kind up to 60 nucleotides (see *Methods*). PolyPhen2, SIFT, FATHMM, MutationAssessor and LRT only classify SNVs, while PROVEAN deals with in-frame but not frameshift indels (see Additional file 3). Furthermore, these tools may be unable to provide some predictions due to lack of information (e.g. when only few homologous sequences exist or remain after their filtering). Therefore, in order to obtain a fair comparison, we removed from the aforementioned test sets those variants that other algorithms were unable to score (Table 6). While PaPI scored every variant, missing rates of the other prediction tools on the three sets ranged from 7.34% to 23%, as reported in Table 7.

**Table 6:** **Filtered test sets**

**Set**	**Damaging**	**Tolerated**	**All**
# 1	5189	3631	8820
# 2	5105	3631	8736
# 3	3618	2553	6171

The three filtered-variant set used for comparison with PolyPhen2, SIFT, Carol, PROVEAN, FATHMM, MutationAssessor and LRT. Test sets are divided by Tolerated and Damaging set.

**Table 7:** **Missing rates on the three unfiltered test sets**

	**% Missing rate**		
	**Set #1**	**Set #2**	**Set #3**
PolyPhen2	7.66	7.68	7.34
SIFT	10.41	10.39	9.8
Carol	11.05	11.44	10.98
PROVEAN	9.85	9.89	9.34
FATHMM	7.64	7.72	7.09
MutationAssessor	9.78	9.72	9.58
LRT	22.99	22.82	22.23
**PaPI**	**0**	**0**	**0**

Missing rates (i.e. algorithm unable to provide prediction) of considered algorithms on the three unfiltered test sets.

**Figure 2:**
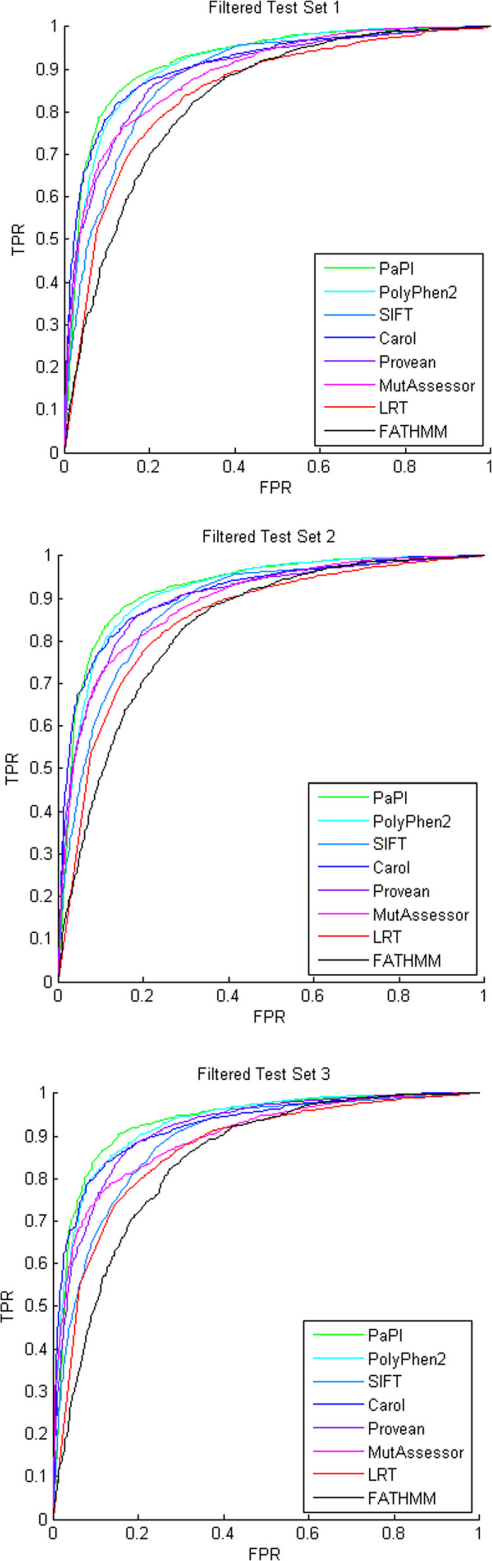
ROC curves comparison between prediction tools. ROC curves of PaPI, PolyPhen2, SIFT, Carol, PROVEAN, FATHMM, LRT and MutationAssessor on the three filtered test sets.

The average AUC and balanced accuracy of PaPI were of 0.926 and 0.864 respectively, reporting an average increase of 1.5 and 3.3 percentage points in balanced accuracy and MCC when compared to the second best predictor (Carol). Negative/positive predictive values and other performance metrics are reported in Table 8. ROC curves of each predictor are reported in Figure 2.

**Table 8:** **Performances of different prediction tools on the three filtered test sets**

**Data Set**	**Tool**	**AUC**	**Balanced Accuracy**	**Sens**	**Spec**	**PPV**	**NPV**	**F-m**	**MCC**
# 1	PaPI	**.9218**	**.8575**	**.8518**	.8631	.8989	**.803**	**.8747**	**.7084**
	Carol	.9120	.8492	.821	**.8774**	**.9054**	.7742	.8611	.689
	Provean	.8938	.8264	.7894	.8634	.892	.7415	.8375	.643
	SIFT	.883	.8142	.7633	.8651	.8899	.7189	.8218	.6185
	PolyPhen2	.9144	.8425	.8503	.8348	.8803	.796	.865	.6806
	FATHMM	.8301	.7517	.6267	.8766	.8789	.6217	.7317	.502
	LRT	.8455	.8249	.8009	.8488	.8833	.749	.8401	.6409
	MutAssessor	.8899	.812	.7578	.8662	.89	.7144	.8186	.6141
# 2	PaPI	**.9246**	**.863**	.8623	.8637	.8989	**.8169**	**.8802**	**.7209**
	Carol	.9121	.8442	.811	**.8774**	**.9029**	.7675	.8545	.6794
	Provean	.8984	.8354	.8074	.8634	.8926	.7613	.8479	.6623
	SIFT	.8836	.8091	.7532	.8651	.887	.7137	.8146	.6094
	PolyPhen2	.9183	.8491	**.8635**	.8348	.8802	.813	.8717	.6957
	FATHMM	.8355	.7603	.6441	.8766	.8801	.6366	.7438	.5187
	LRT	.8506	.8317	.8147	.8488	.8834	.7651	.8477	.656
	MutAssessor	.8923	.8134	.7606	.8662	.8888	.7202	.8197	.6178
# 3	PaPI	**.9332**	**.8721**	**.8751**	.8692	.9046	**.8308**	**.8896**	**.7398**
	Carol	.9239	.8551	.8187	**.8915**	**.9145**	.7763	.8639	.7004
	Provean	.9159	.8444	.8156	.8731	.9011	.7697	.8562	.6797
	SIFT	.8911	.8166	.759	.8743	.8953	.7191	.8215	.6238
	PolyPhen2	.9303	.8542	.8729	.8355	.8826	.8226	.8777	.7068
	FATHMM	.8436	.7643	.6410	.8876	.8899	.6356	.7452	.527
	LRT	.8682	.8408	.8289	.8527	.8886	.7786	.8577	.6744
	MutAssessor	.8988	.8273	.7772	.8774	.8998	.7354	.8341	.6449

Comparison of PaPI, PolyPhen2, SIFT, Carol, PROVEAN, FATHMM, LRT and MutationAssessor on the three test sets filtered for unpredictable variants by the other prediction tools. Area under the curve (AUC), balanced accuracy (sensitivity/2 + specificity/2), sensitivity (Sens), specificity (Spec), Positive Predictive Value (PPV), Negative Predictive Value (NPV), F-measure (F-m) and Matthews correlation coefficient (MCC) are reported for each method. Highest values for each set are marked in bold.

### PaPI exploits pseudo amino acid composition substitution patterns for disease-related variant prediction

We hereby show the case of a disease-related variant for which the RF is the solely to correctly assign the right prediction in contrast to PolyPhen2, SIFT and the other cited tools. This example shows therefore how the RF can positively contribute to a correct variant evaluation by exploiting pseudo amino acid composition substitution patterns in specific protein-coding regions.

#### GUCY2C - Asp387Gly

Romi *et al.* [40] identified a single mutation p. (Asp387Gly) in the guanylate cyclase 2C (GUCY2C) transmembrane receptor causing meconium ileus (MI), an intestinal obstruction in newborns. GUCY2C has an extracellular domain that is activated by ligands (guanylin and related peptide uroguanylin or E.coli heat-stable enterotoxin STa). The p. (Asp387Gly) mutation is within an essential region of its extracellular ligand-binding domain and is adjacent to seven other pivotal amino acids for the ligand binding [41]. The resulting significant reduction of ligand-binding leads to a reduction in guanylate cyclase activity and activates a signalling cascades that finally leads to MI.

The GUCY2C extracellular domain belongs to the periplasmic binding protein-like I superfamily domain and corresponds to the extracellular ligand-binding receptor, IPR001828 in InterPro database [42]. The same domain is shared by other 157 human proteins (including the one encoded by GUCY2C). Among the disease variant set used for RF training, 242 disease variants belonging to the IPR001828 domain and related to 7 proteins are present. We suppose that the RF learned the model of substitution patterns in the extracellular domain of these proteins and therefore it was able to assign the correct prediction for p. (Asp387Gly) in the GUCY2C encoded protein.

### PaPI leads to right prediction in case of PolyPhen2 and SIFT conflict

Here we report an example that shows how PaPI can correctly classify variants for which PolyPhen2 and SIFT are discordant in prediction.

Tchernitchko *et al.* [43] compared PolyPhen and SIFT considering several variants known to be responsible for affecting the products of hemoglobin and glucose-6-phosphate dehydrogenase genes, leading to several forms of sickle cell anemia and G6PD deficiency, respectively. In the results in that paper, PolyPhen and SIFT had discordant predictions on ten pathogenic variants, for which experimental evidence was reported. We therefore run PaPI on the same variant set and we correctly classified all of them as damaging. Five variants were predicted both by PolyPhen2 and SIFT (thanks to updated versions now available) as damaging while for the other five variants there were still discordant predictions between these tools (Table 9). For these cases, the RF vote allowed obtaining the right class assignment. Notably, SIFT is able to assign the right class for each variant as well, despite we show that RF and SIFT have the lowest concordance rate in prediction in case of PolyPhen2 conflict (see Additional file 1: Figure S2). The complete variant list is reported in Additional file 4.

**Table 9:** **Examples of known disease-related variants**

**Gene**	**Protein**	**PP2**	**SIFT**	**RF**	**Related Phenotype**	**PaPI**
HBB	p.E7V	B (0.002)	D (0.01)	D (0.891)	Sickle cell anemia	D (0.901)
	p.E122Q	B (0.007)	D (0.01)	D (0.975)	Severe sickle cell syndromes	D (0.975)
	p.E122K	B (0.109)	D (0.0)	D (0.96)		D (1.0)
	p.E7K	B (0.006)	D (0.01)	D (0.94)		D (0.94)
G6PD	p.S188F	B (0.039)	D (0.04)	D (0.988)	G6PD deficiency	D (0.988)

Known disease-related variants reported by Tchernitchko *et al.* for which occurs a different outcome in prediction by PolyPhen2 (PP2) and SIFT. For these cases, the RF is able to vote for the right class leading PaPI to the correct prediction as well. In brackets the score for each variant predictor is reported. B = tolerated, D = damaging.

### Web accessible tool

PaPI software is freely available online (http://papi.unipv.it) as a web accessible tool.

The user interface allows to submit a single variant or to perform queries in bulk by uploading a plain text file with a list of variants.

Users can choose between the RF and LR model. Although we showed LR is less accurate than RF, it is faster and can be used for a quick response.

Two different gene annotation models are available (RefSeq and GENCODE) and a variant score is given for each different transcript.

Results are reported in a tab-delimited text file and can be sent by email: PaPI prediction (damaging or tolerated) along with its confidence score plus prediction/scores of RF/LR, PolyPhen2 and SIFT. Each variant comes with information about transcript, gene, type (missense, synonymous, frameshift etc.) and evolutionary conservation scores. Prediction runtime takes, in average, between 0.3 and 0.7 seconds per variant.

## Conclusions

We developed a new method, called PaPI, to classify and score human coding variants potentially leading to functional alterations of related proteins. Since the algorithm has been trained on HGMD database, which is intended for the analysis of human Mendelian diseases, we expect PaPI to be more accurate for such class of diseases.

The main novelty of the approach is the introduction of features based on the difference in pseudo amino acid composition between snippets of wild and altered protein sequences where coding variants occur. Hydrophobicity and hydrophilicity pairwise relationships between amino acids are encoded by these features. Evolutionary conservation scores and quantitative descriptors at the whole protein level were included in the feature set as well. A RF classifier was trained on these features to mine disease and neutral pseudo amino acid composition substitution patterns and classify unseen coding variants into damaging or tolerated class.

Despite it has been shown that the combination of variant classifiers is not always beneficial [44], we showed that the implemented voting strategy between PolyPhen2, SIFT and our RF model improves performances in terms of area under the curve, accuracy and other reported metrics in comparison to the ones of each predictor alone. Considering only those variants that PolyPhen2 and SIFT are unable to predict, PaPI maintains high performances thanks to the RF model. Moreover, it has to be noted that in case of prediction by both PolyPhen2 and SIFT, PaPI is biased toward sequence conservation, because of the majority voting system between the RF and these two tools [45].

We compared PaPI with other variant prediction tools (PolyPhen2, SIFT, Carol, PROVEAN, FATHMM, MutationAssessor, LRT) and we showed that PaPI performances were the highest on the data sets used. Notably, PaPI is able to score any variant, including the ones that the other mentioned methods were unable to predict.

We have reported an example for which the RF model is the only algorithm that predicts the correct class, thanks to its capability of exploiting potential disease-related pseudo amino acid composition substitution patterns such as protein ligand-binding domains. We also showed several examples where the RF model vote leads to a correct prediction, in case of conflict between PolyPhen2 and SIFT.

To our knowledge, PseAAC has never been used in variant prediction. We are confident that the algorithm can be further improved by optimizing other parameters (e.g. length of sequence snippets surrounding variants) or by exploring other PseAAC descriptors (e.g. including amino acid side chain mass property).

## Methods

**Figure 3:**
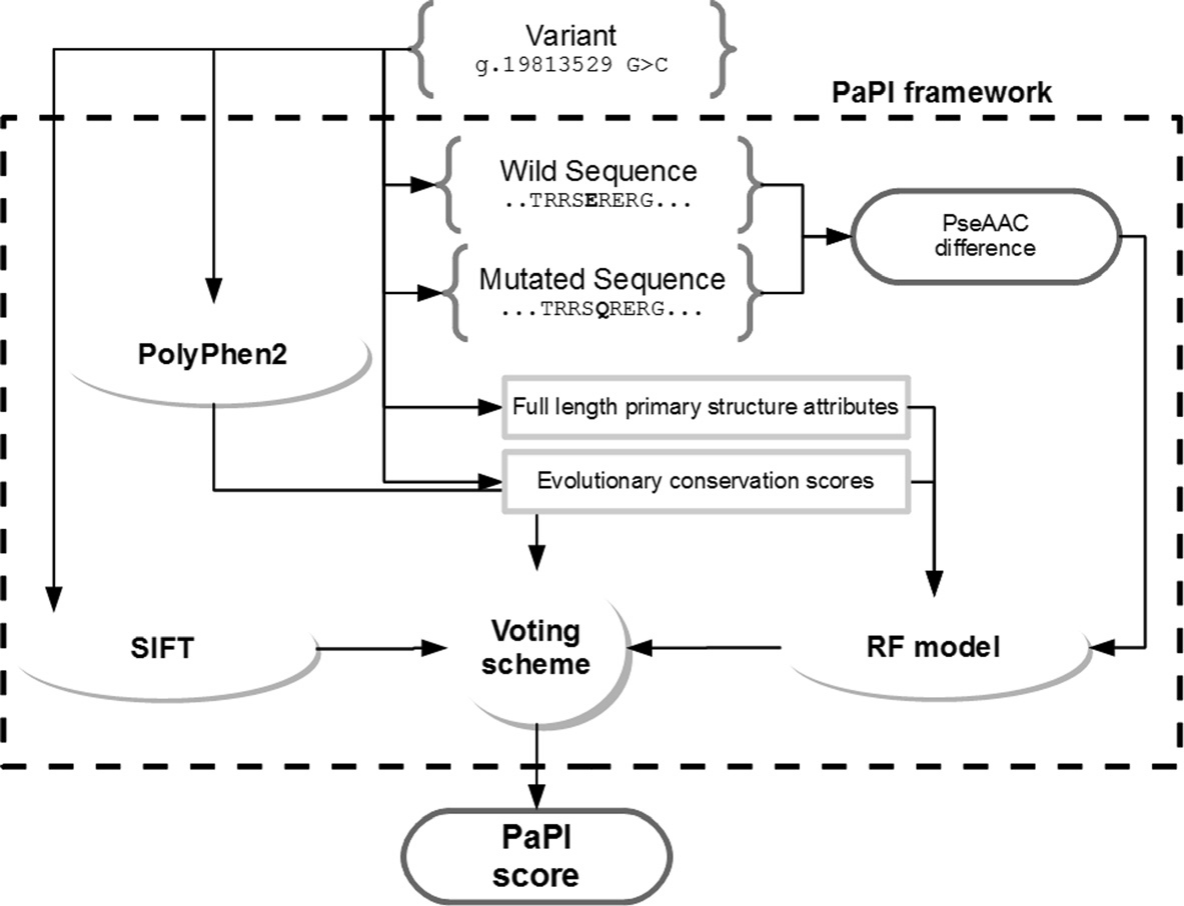
Feature encoding scheme. A genomic variant is translated into wild and mutated amino acid sequences. The difference in terms of PseAAC features is computed and is given as input to the trained RF model along with evolutionary conservation scores and several full-length protein attributes. PolyPhen2, SIFT and RF results are finally combined together to obtain the final PaPI class and score.

PaPI is an ensemble classifier consisting of a voting scheme that includes a RF classifier, PolyPhen2 and SIFT. The RF model have been trained on PseAAC differences of mutated and wild protein sequences, evolutionary conservation scores and several full-length protein attributes. Figure 3 depicts the workflow through which a new variant is classified and assigned a score, representing its risk of being protein damaging.

### Psuedo amino acid composition

An amino acid sequence can be represented by a set of discrete numbers mapping the patterns of its amino acid physico-chemical properties into a fixed number of features.

Traditional amino acid composition approach has been widely used in predicting protein structural class [46,47] and it merely records amino acids frequencies in a protein sequence.

PseAAC adds a number of position-related features and therefore it reflects both compositional and sequential order. We utilized, in particular, amphiphilic PseAAC, based on normalized hydrophobicity and hydrophilicity [31].

**Figure 4:**
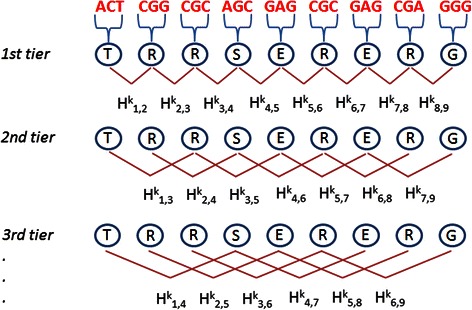
Amphiphilic PseAAC representation. This is a diagram shows how the correlation factors H^k^, based on amino acid hydrophobicity (k = 1) and hydrophilicity (k = 2), vary in each tier by coupling residues at different distances.

In brief, given a protein sequence *P* with *L* amino acid residues:

$$ \mathrm{P}={\mathrm{A}}_1{\mathrm{A}}_2{\mathrm{A}}_3{\mathrm{A}}_4{\mathrm{A}}_5{\mathrm{A}}_6 \dots {\mathrm{A}}_{\mathrm{L}} $$

it is possible to convert it into a finite set of number *P’*

$$ \mathrm{P}'=\left[{\mathrm{p}}_1,\ {\mathrm{p}}_2,\ {\mathrm{p}}_3,\ {\mathrm{p}}_4,\ {\mathrm{p}}_5,\dots,\ {\mathrm{p}}_{20},\ {\mathrm{p}}_{20+1},\dots,\ {\mathrm{p}}_{20+2\lambda}\right] $$

where the first 20 numbers are functions of the frequencies of the 20 amino acids within *P* and the remaining 2λ are a set of correlation factors that reflect different hydrophobicity and hydrophilicity distribution patterns along a protein chain. Correlation factors are given by coupling the most contiguous residues whose contiguity condition varies according the considered tier (see Figure 4). The maximum number of tiers corresponds to λ. Coupling is then given by the hydrophobicity and hydrophilicity correlation functions.

1 $$ {{\mathrm{H}}^1}_{\mathrm{i},\mathrm{j}} = {\mathrm{h}}^1\left({\mathrm{A}}_{\mathrm{i}}\right) \cdot p\ {\mathrm{h}}^1\left({\mathrm{A}}_{\mathrm{j}}\right),\ {{\mathrm{H}}^2}_{\mathrm{i},\mathrm{j}} = {\mathrm{h}}^2\left({\mathrm{A}}_{\mathrm{i}}\right) \cdot p\ {\mathrm{h}}^2\left({\mathrm{A}}_{\mathrm{j}}\right) $$

where h^1^ (A_i_) and h^2^ (A_i_) are, respectively, the hydrophobicity and hydrophilicity values for the i^th^ (i = 1,2,…, *L*) amino acid in *P*. Correlation functions are summed over each λ-tier and the 20 + 2λ coupling factors are given\

2 $$ {p}_u=\left\{\frac{\frac{f_u}{{\displaystyle {\sum}_{i=1}^{20}{f}_i+w{\displaystyle {\sum}_{j=1}^{2\lambda }{\tau}_j}}},1\le u\le 20}{{\displaystyle \kern1em {\sum}_{i=1}^{20}{f}_i+w{\displaystyle {\sum}_{j=1}^{2\lambda }{\tau}_j}}},20+1\le u\le 20+\lambda \right. $$

where f_i_ are the normalized frequencies of the possible 20 amino acids in *P*, τ_j_ is the sum of the j-tier correlation functions and *w* is a weight factor.

### Feature set

The features utilized for RF and LR training can be divided into three groups: (a) PseAAC, (b) full-length primary sequence attributes, and (c) evolutionary conservation scores. The three feature groups are described as follows.

#### PseAAC

**Figure 5:**
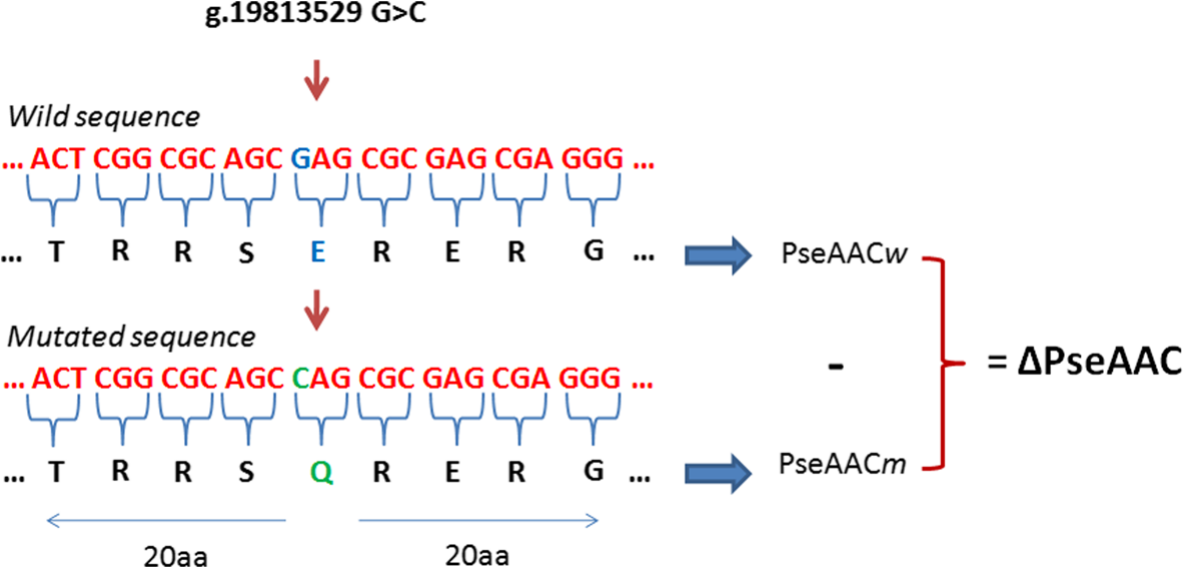
Example of PseAAC variant feature encoding. A genomic variant is translated into the relative wild and mutated amino acid sequences. PseAAC for both wild and mutated protein snippets are computed and the differences between each PseAAC term makes the PseAAC feature set.

Given a genomic variant overlapping a protein, we first generated the altered protein sequence in according to the coding frame, we then considered the 20 amino acid residues upstream and downstream the first mutated amino acid forming a snippet of 41 amino acid residues. The same procedure is followed in the case of the corresponding wild type protein sequence. Amphiphilic PseAAC is then computed by PseAAC-builder [48] for both wild and mutated snippets. The variant-sequence features are finally encoded as the element-wise difference of wild and mutated PseAAC vectors (see Figure 5). Note that even if the method allows theoretically dealing with amino acid sequence changes of any length, only insertions/deletions up to 20 amino acids (60 nucleotides) were considered for PseAAC model training.

We chose two 20 amino acid flanking regions for two reasons. First, since the features are encoded by PseAAC differences, considering large sequence portions (e.g. the whole primary structure) could introduce noise and dilute the PseAAC difference information content, especially in case of single amino acid substitution, where both positional and composition change would be minimal. Second, we considered that protein short functional regions, such as short linear motifs, which play a pivotal role in protein interactions, range from 3 to 11 amino acids in length [49]. Changes in their flanking regions could severely alter the protein function as well [40,50]. We therefore assumed that 20 amino acids constitute a reasonable window size to encompass possible short functional motifs and their flanking regions.

#### Full length primary sequence attributes

We included in the RF model three features related to variant position and protein length. First, we considered the difference and the ratio between mutated and wild amino acid sequence lengths. In other words, we measured the number of possible lost/inserted amino acids caused by the variant. Second, we considered the position of the variant in the amino acid sequence normalized by the protein length (e.g. 0.9 for a 100 amino acids long protein and its mutated amino acid at the 90th position). This feature reflects the fact that some kind of variants affecting the initial part of the primary sequence may have a huge damaging potential for the whole protein (e.g. a stop-causing variant).

#### Evolutionary conservation scores

Gerp++ [18], PhyloP [19] and Siphy [20] were chosen because they apply different and complementary methods to weight nucleotide conservation among different species. In case of indels, the following policy was adopted: in case of deletion we took the highest score among the deleted nucleotide bases; in case of insertion we took the highest score between the two reference bases where insertion occurs.

### Parameter tuning

The implemented RF model is based on Weka libraries [51]. We tuned RF model parameters by running an independent 10-fold cross validation on each of the generated training sets. The considered parameters were four, two related to the RF (number of trees and number of features per node) and two related to the PseAAC (λ and *w*). Parameter details are shown in Table 10. Note that the 41 amino acid snippet length used to compute PseAAC is fixed and it was not included in the optimization parameters. For each training set, we obtained the same optimal set of parameters, w = 0.1, λ = 12, number of trees = 250 and number of features per node = 2.

**Table 10:** **Parameter values used for RF model tuning**

**Parameter**	**Used in**	**Values**
# trees	RF	5, 10, 50, 100, 150, 200, 250, 300, 350
# features per node	RF	int (log (# trees) +1), 2, 4
λ	PseAAC	4, 8, 12, 16, 20
w	PseAAC	0.5, 0.1

List of parameters and relative values used for the optimization of the RF model on training sets.

According to the PseAAC representation, λ determines the number of positional features (if λ = 0, we have the traditional amino acid composition representation). In the amphiphilic PseAAC, features are 20 (frequency related) + 2*λ (positional). As a consequence, the total number of features varies according to λ, from a minimum of 30 (24 for PseAAC, 3 for quantitative attributes and 3 evolutionary conservation scores) to a maximum of 66 (60 for PseAAC + 3 for quantitative attributes + 3 evolutionary conservation scores). Thus, with λ = 12, our RF model uses 50 features (44 + 3 for quantitative attributes + 3 evolutionary conservation scores). Being λ responsible for 4 to 60 features in the RF model, the feature selection process stands implicitly in the λ parameter tuning. The selected model includes 50 features and it is trained on datasets including tens of thousands variants (from 31601 to 44888, as shown in Table 2). Since the number of samples is much greater than the number of features, we did not proceed with a further feature selection.

Amino acid sequences shorter than λ + 1 cannot be represented with PseAAC. This issue, nevertheless, can happen only in the case of a coding mutation that introduces a premature stop-codon at the beginning of the protein: this is the case of stop-gain variants; these mutations are automatically labeled as deleterious. It has to be noted that, considering λ = 12, only 438 mutated sequences (out of about 204 K of the overall dataset) were too short to be represented by this PseAAC model.

### Data sets

We obtained positive (damaging) variants from the HGMD (updated to May 2013). Variants were annotated by ANNOVAR [52] using the RefSeq gene model. All non-coding variants, as well as variants reported with a frequency higher than 5% in the total of 1092 samples from 1TGP (April 2012 release) were filtered out. Negative (tolerated) variants were extracted from the aforementioned release of 1TGP and from ESP (6500si release) retaining only variant at frequency higher than 0.05. Non-coding and synonymous variants were filtered out by ANNOVAR. Each variant was then processed by the PaPI annotation framework in order to build the relative feature set (see Additional file 5: S1).

The original variant dataset consisted of 204021 coding SNVs and indels, distinguished by transcript and filtered out for synonymous SNVs. Stratification for descriptive protein alteration to the primary structure unveiled a significant proportion (about 44%) of frameshift indels or stop-causing/disrupting variants in the damaging set in comparison to the tolerated one (about 3%), as reported in Table 1.

One can suppose these variants should be treated as deleterious *a priori*: the proportion showed above is in accordance with this hypothesis. Including these variants in our data set would introduce a severe classification bias, due to the aforementioned disproportion. Therefore we randomly assembled three quasi-balanced training (70%) and test (30%) sets (Table 2) without considering these types of variants for the evaluation and comparison steps, but we trained the final RF model (available online) on the whole unfiltered dataset. Indeed, PaPI is capable to score stop-causing/disrupting and frameshift variants as well. The three test sets have been used to measure the performances of the RF and LR (Table 3). In order to compare RF, PolyPhen2, SIFT and PaPI (RF + PolyPhen2 + SIFT) on the three test sets we further filtered out the variants that PolyPhen2 and/or SIFT were not able to classify (see Additional file 1: Table S2).

### Comparison data set

**Figure 6:**
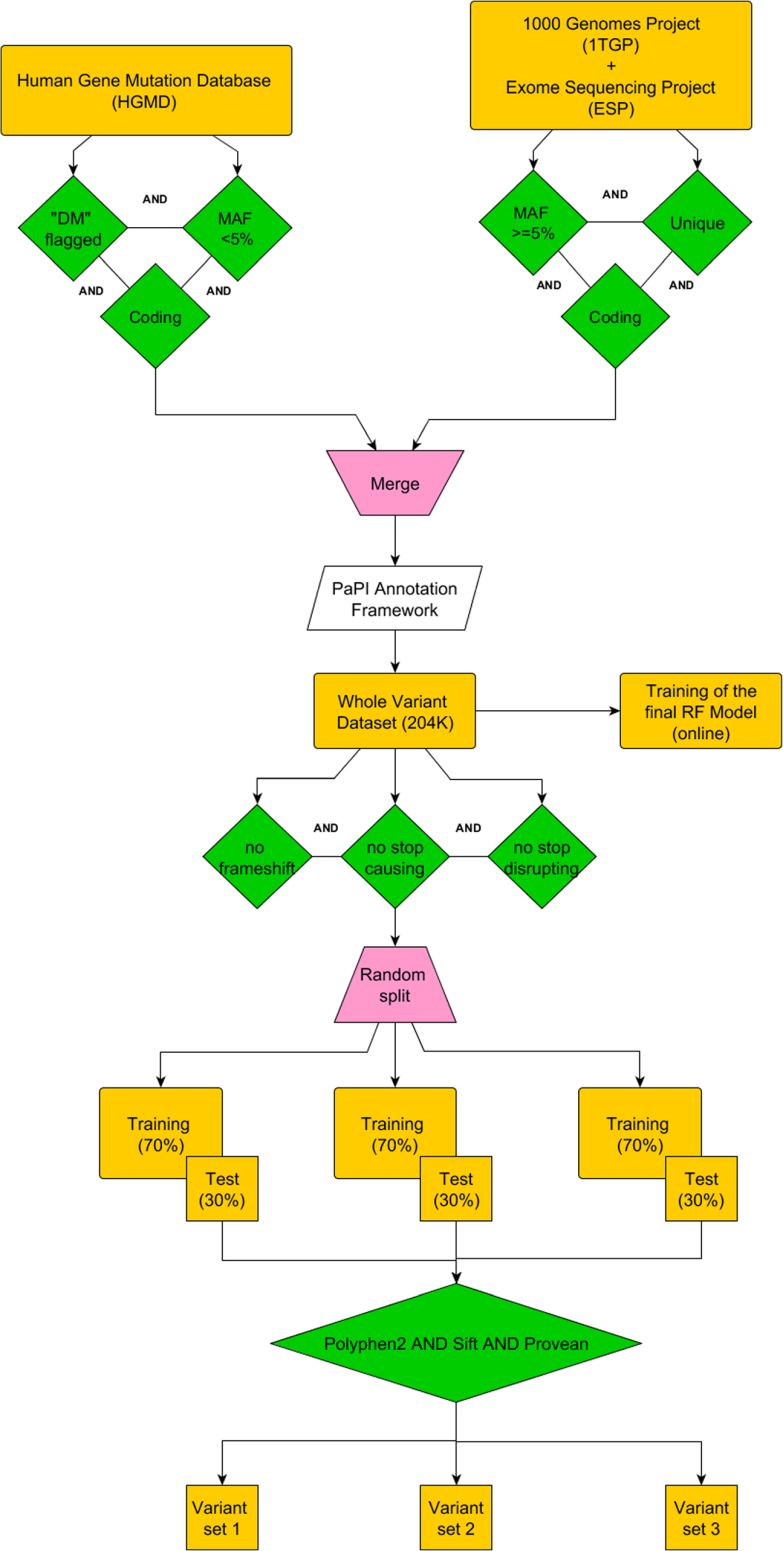
Data set workflow. Workflow representing the data set selection. Variants from HGMD, 1TGP and ESP were filtered basing on coding, frequency and non-overlapping (unique) variants among the different data sources. In order to evaluate and compare the performances of the variant predictor tools, variants were further filtered for frameshift, stop-disrupting, stop-causing and variants not predictable for the other algorithms.

PolyPhen2, SIFT and the other compared predictors only classify SNVs. Furthermore, these tools may be unable to provide any prediction for lack of information (e.g. when only few homologous sequences exist or remain after their filtering). To avoid any bias that could favor PaPI, we removed from the aforementioned test sets all the variants that other algorithms were unable to score (Table 6). The whole data set filtering and processing workflow is shown in Figure 6. Note that we grouped all the different transcript-variants for each variant in the same set, i.e. all the mutated protein isoforms for a variant were either all in the training or in the test set. This procedure assured that very similar instances were not present in both training and test sets.

### Voting scheme

The RF model score is computed as the posterior probability of the class. For each instance, the RF model will provide a probability score for damaging class and its complement to one for the tolerated class. The instance is thus considered damaging if the related score is equal or larger than 0.5, and a tolerated variant otherwise.

SIFT and PolyPhen2 provide scores in the [0, 1] interval, and the thresholds *t*_*s*_ to separate damaging and tolerated variants are 0.447 and 0.05 respectively. We needed to standardize both SIFT and PolyPhen2 scores in order to compare them with our RF model score. We thus remapped SIFT and PolyPhen2 results by forcing scores < *t*_s_ in the [0, 0.5 [interval, and scores > *t*_s_ in the [0.5, 1] interval according the following standardization

$$ \mathrm{A}=\left(\left(\mathrm{A}'- \min \left(\mathrm{A}'\right)\right)/\left( \max \left(\mathrm{A}'\right)- \min \left(\mathrm{A}'\right)\right)\right)*\left( \max \left(\mathrm{A}\right)- \min \left(\mathrm{A}\right)\right)+ \min \left(\mathrm{A}\right) $$

Where A’ is the score in the original interval and A is the score mapped to the new interval, while min/max (A) and min/max (A’) are the minimum and maximum scores of the new and original interval, respectively.

A majority voting scheme is then applied when each of the three models provides a prediction. That is, in case of conflict between two tools, the vote on class prediction of the third is determinant for the final class assignment (damaging or tolerated). The normalized score of the most confident tool (distance from decision threshold) is taken as the final score. If PolyPhen2 or SIFT are not able to provide a prediction, the most confident normalized score between the remaining two algorithms leads class and score assignment. Finally, in case both PolyPhen2 and SIFT are not able to provide a prediction, only the RF model is used.

Usually the more tools are combined, the smaller is the number of the cases that all of them can predict [45]. However, PaPI is not affected by this limitation since the RF model and the policy used allow obtaining a prediction even when PolyPhen2 and/or SIFT do not.

## Additional files

Additional file 1:
**Supplementary Tables and Figures.**

Additional file 2:
**Variant prediction tools: a brief description.**

Additional file 3:
**Type of variants predicted.**

Additional file 4:
**List of known** **disease-related** **variants by Tchernitchko et al.** Known disease-related variants reported by Tchernitchko *et al.* for which a different outcome in predictions by the outdated version of PolyPhen and SIFT occurred. 
Additional file 5:
**Annotation procedure.**

## Abbreviations

1TGP: The 1000 Genomes Project
ESP: NHLBI GO Exome Sequencing Project
HGMD: Human Gene Mutation Database
PseAAC: Pseudo Amino Acid Composition
RF: Random Forest
LR: Logistic Regression
SNV: Single Nucleotide Variants
AUC: Area Under the Curve
MCC: Matthews Correlation Coefficient
