# Supplementary material for: PaPI: pseudo amino acid composition to score human protein-coding variants
Source: BMC Bioinformatics. 2015 Apr 19;16:123. doi: 10.1186/s12859-015-0554-8 (PMC4411653; doi:10.1186/s12859-015-0554-8)
Supplement: Additional file 1: — Supplementary Tables and Figures. [file 12859_2015_554_MOESM1_ESM.pdf]

Additional file 1

Supplementary Tables

Tool	Test Set	AUC	Balanced accuracy	F-m	MCC
RF – PseAAC only	# 1	.885	.816	.816	.628
	# 2	.888	.821	.821	.637
	# 3	.892	.827	.827	.65

Table S1. Performances of the RF trained only using PseAAC features, measured on the three unfiltered test sets. Area under the curve (AUC), balanced accuracy (sensitivity/2 + specificity/2), F-measure (F-m) and Matthews correlation coefficient (MCC) are shown.

Set	Damaging	Tolerated	All
**# 1**	5316	5153	10469
**# 2**	5323	5153	10476
**# 3**	3763	3642	7405

Table S2. The three filtered variant set used to measure performances of RF, PolyPhen2, SIFT and PaPI. Test sets have been divided by Tolerated and Damaging set. Variants on multiple transcripts have been counted once.

Supplementary Figures

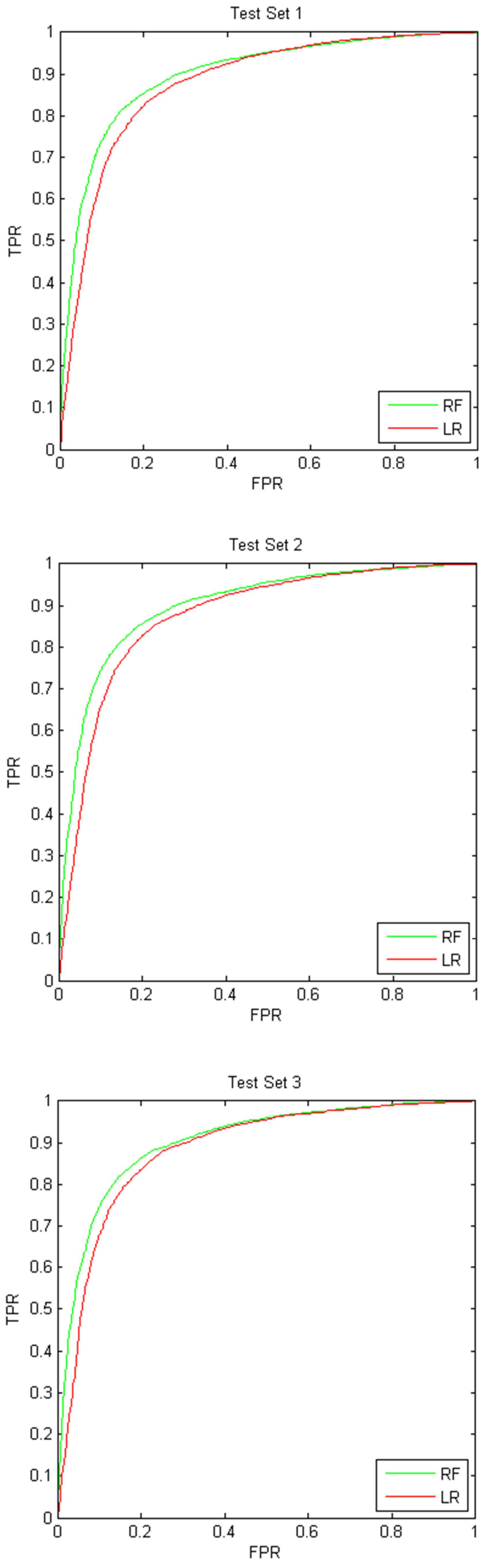

Figure S1. ROC curves of Logistic Regression (LR) and Random Forest (RF) on the three unfiltered variant test sets.

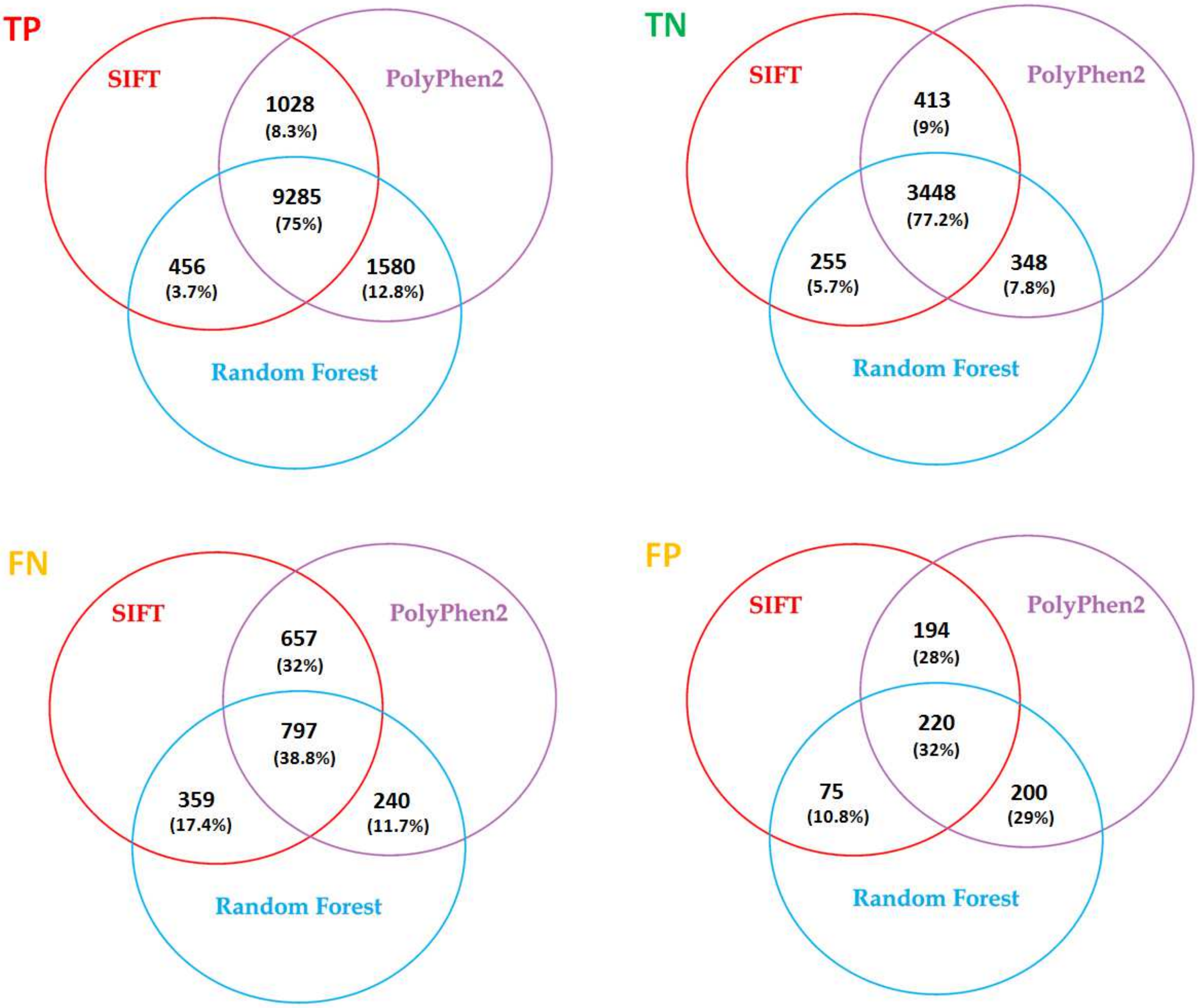

Figure S2. Venn diagrams showing contingencies in terms of prediction agreement between the Random Forest, SIFT and PolyPhen2 on the whole variant test set where both PolyPhen2 and SIFT hold a prediction. P=Positive (Damaging), N=Negative (Tolerated).

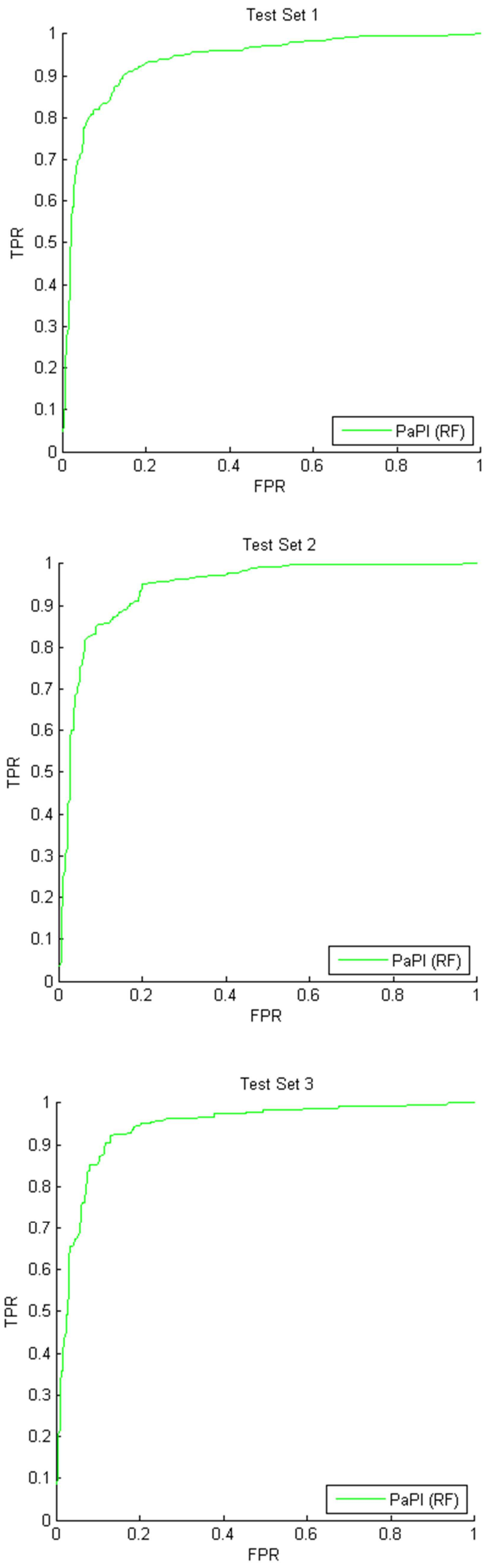

Figure S3. ROC curves of PaPI on the three variant test sets after retaining those variants unpredictable for both PolyPhen2 and SIFT. For these cases PaPI coincides with RF.
