# Supplementary material for: PaPI: pseudo amino acid composition to score human protein-coding variants
Source: BMC Bioinformatics. 2015 Apr 19;16:123. doi: 10.1186/s12859-015-0554-8 (PMC4411653; doi:10.1186/s12859-015-0554-8)
Supplement: Additional file 2: — Variant prediction tools: a brief description. [file 12859_2015_554_MOESM2_ESM.pdf]

Additional file 2

Brief description of the other prediction tools used

PolyPhen2 trains a naïve Bayes classifier on various multiple sequence alignments methods (MSA) of homologous proteins and protein structure-based features sets (e.g. accessible surface area propensity for buried residues change, crystallographic beta-factor etc.). In this work, we utilized PolyPhen2-HumVar model. PolyPhen2 is not able to provide predictions or give wrong predictions in case of (a) mutations outside conserved domains and (b) MSA lacks of homologous protein sequences [1-2].

SIFT computes a combined score derived from position-specific scoring matrices with a Bayesian method based on Dirichlet priors and the distribution of amino acid residues observed at a given position in the MSA of homologous proteins. SIFT suffers the same kind of limitations of PolyPhen2.

Carol is an ensemble of PolyPhen2 and SIFT and uses a weighted *Z* method to combine their individual probabilistic scores. It predicts only non-synonymous SNVs for which both PolyPhen2 and SIFT are able to give a prediction.

PROVEAN uses an alignment-based score to measure the change in sequence similarity of a query sequence to a homologous protein sequence before and after the introduction of an amino acid variation. It is able to deal with non-synonymous SNVs and in-frame DIVs. As all the other methods that are based on MSA, it can fail and not provide any prediction because of the lack of homologous protein sequences.

FATHMM (Functional Analysis Through Hidden Markov Models) uses MSA based on protein homologous sequences to build an Hidden Markov Model where sequence conservation is interrogated through the internal match states of the model. We have used the unweighted version of FATHMM for the comparison with the other prediction tools.

MutationAssessor uses hierarchical clustering to identify specificity residues and the corresponding optimal division into subfamilies given an MSA of large numbers of homologous sequences. Then it computes the functional impact score basing on conservation among all sequences of a protein family and subfamily specificity. Mutation Assessor scores and predictions have been extracted by dbNSFP v2.4.

LRT is based on a comparative genomics data set of 32 vertebrate species and applies a likelihood ratio test (LRT) to compare the null model that each amino acid is evolving neutrally to the alternative model of evolution under negative selection. LRT scores and predictions have been extracted by dbNSFP v2.4.
