# Supplementary material for: PaPI: pseudo amino acid composition to score human protein-coding variants
Source: BMC Bioinformatics. 2015 Apr 19;16:123. doi: 10.1186/s12859-015-0554-8 (PMC4411653; doi:10.1186/s12859-015-0554-8)
Supplement: Additional file 5: — Annotation procedure. [file 12859_2015_554_MOESM5_ESM.pdf]

Additional file 5

S1. PaPI Annotation framework

Each genomic variant (SNVs and indel) is annotated by one of the available gene models (RefSeq or GENCODE). Non coding RNAs and ORF genes were excluded. Selenoproteins, for which the UGA-stop-codon in the middle of their coding regions codifies for selenocysteine were included: notably, we observed that no one of the other mentioned prediction tools cited in this paper is able to correctly deal with these particular genes, even if several disorders involving changes in selenoprotein structure, activity or expression have been reported (Bellinger FP *et al*). Therefore, only variants that overlap the identified coding regions of the above gene models are considered for downstream analysis. All possible transcripts for which the variant is coding are retrieved, and features are computed for each transcript. In particular, for PhyloP and Gerp++ positional scores that can involve more than one base change, (a) in case of deletion/indel the maximum score between the deleted bases is taken, while (b) in case of insertion the maximum score between the two neighbour genomic positions is taken. Siphy score is included only in case of missense SNVs using dbNSFP (v2.1) datasource (Liu X *et al*). DbNSFP database was used to retrieve SIFT and PolyPhen2 pre-computed prediction scores as well.

REF.

[1] Bellinger FP, Raman AV, Reeves MA, Berry MJ. Regulation and function of selenoproteins in human disease. Biochem J. 422(1), 11-22 (2009).

[2] Liu X, Jian X, and Boerwinkle E. dbNSFP v2.0: A Database of Human Nonsynonymous SNVs and Their Functional Predictions and Annotations. Human Mutation 34, E2393-E2402 (2013)
